# Hyptolide induces ER stress-mediated cell death and enhances GSK3β-regulated cisplatin chemosensitivity in ovarian cancer

**DOI:** 10.1186/s13048-025-01712-4

**Published:** 2025-06-12

**Authors:** Yusi Luluk Rahmania, Person Pesona Renta, Damar Nurwahyu Bima, Yu-Shan Lin, Ngoc Thang Nguyen, Pin-Yu Wang, Meiny Suzery, Wen-Tai Chiu

**Affiliations:** 1 Department of Biomedical Engineering, National Cheng Kung University, Tainan, 701 Taiwan; 2 Department of Marine Science, Faculty of Agriculture, University of Bengkulu, Bengkulu, 38371 Indonesia; 3 Department of Chemistry, Faculty of Science and Mathematics, Diponegoro University, Kota Semarang, 50275 Indonesia; 4 International Center for Wound Repair and Regeneration, National Cheng Kung University, Tainan, 701 Taiwan; 5 Medical Device Innovation Center, National Cheng Kung University, Tainan, 701 Taiwan

**Keywords:** Hyptolide, Ovarian cancer, Chemoresistant, ER stress, GSK3β, β-catenin, E-cadherin, MET

## Abstract

**Background:**

Ovarian cancer is a highly prevalent cancer among women with a high risk for relapse and drug resistance. Seventy eight percent of women diagnosed with ovarian cancer live for at least one year after diagnosis. Hyptolide, a natural compound, has been shown to act as an anti-inflammatory and antibacterial agent, and latest research shows that it acts as an anticancer agent. These properties indicate that hyptolide may be a potential treatment option for ovarian cancer, including chemoresistant cases; however, its effects in chemoresistant ovarian cancer have not yet been demonstrated, and the mechanisms underlying its induction of cell death remain unclear.

**Results:**

We found that hyptolide inhibited cell viability in ovarian cancer cell lines, regardless of their chemoresistance, and these effects were mediated by ER stress and the activation of GRP78 and ATF6. Combined treatment of cisplatin-resistant cell lines with hyptolide and cisplatin demonstrated a synergistic effect, enhancing apoptosis. Additionally, the reversal of chemoresistance with hyptolide treatment was mediated by β-catenin cytoplasm translocation leading to E-cadherin expression, ultimately promoting mesenchymal-epithelial transition.

**Conclusion:**

Our findings suggest that hyptolide induces ER stress-mediated cell death and overcomes cisplatin chemoresistance in ovarian cancer cells, supporting its potential use as a chemotherapeutic agent.

**Supplementary Information:**

The online version contains supplementary material available at 10.1186/s13048-025-01712-4.

## Introduction

Ovarian cancer is the seventh most common malignancy in women worldwide [1]. Globally, an estimated 239,000 new cases of ovarian cancer and 152,000 deaths are reported annually. In 2020, 313,959 new cases of ovarian cancer were recorded globally with an age-standardized incidence rate of 6.6 per 100,000 [2]. It is the most fatal gynecological cancer, as it is often asymptomatic in the early stages [3]. Seventy percent of ovarian cancers are not diagnosed until they progress to stage III or IV, resulting in a 47.5% survival rate [4, 5]. The treatments administered to patients with ovarian cancer often depend on factors such as the cancer stage [6], with surgical treatment and platinum-based chemotherapies regarded as the gold standard for ovarian cancer therapy [7]. Platinum-based chemotherapies often result in an initial positive response; unfortunately, there is a tendency for disease relapse and drug resistance [8, 9]. Given the limitations of existing treatments, promising novel therapies are urgently needed. [10].

In the modern era of drug development, natural compounds have proven to be an unmatched source of anticancer drugs [11]. Hyptolide is a natural compound extracted from *Hyptis pectinata* (L.) Poit. The highest hyptolide content is found in the leaves [12]. The compound has an α,β unsaturated lactone structure and has been recently identified as a potential anticancer agent [13]. The anticancer activity of hyptolides has been demonstrated in several previous studies. Hyptolide effectively suppresses the growth of MCF-7 and T47D breast cancer cells [14]. The cytotoxicity of hyptolide against cancer cells was also verified in the following cell lines: human metastatic breast cancer MDA-MB-435, human glioblastoma SF-295, human promyelocytic leukemia HL-60 [15], and human triple-negative breast cancer MDA-MB-231 cells [12].

The endoplasmic reticulum (ER) is a vital organelle for the production, folding, and modification of transmembranes and secreted proteins. Although this process is tightly regulated, various external influences and internal cellular processes can disrupt proper protein folding in the ER, resulting in ER stress characterized by the accumulation of misfolded or unfolded proteins [16]. Previous studies have established a correlation between ER stress and several cell functions, including apoptosis, autophagy, and chemoresistance [17–19]. The close relationship between ER stress with apoptosis in cancer cells were identified as a mechanism underlying the effects of hyptolide in cancer treatment in the current study. Epithelial-mesenchymal transition (EMT) is highly correlated with cancer progression and fibrosis. In cancer cells, EMT is a fundamental process in cancer metastasis, invasion, and drug resistance [20]. Among EMT activators, the WNT/β-catenin pathway emerged as a versatile modulator [21]. Cell surface receptors bind with WNT ligands to activate Disheveled protein, which forms a complex with other proteins like GSK3β, Axin2, and APC to release β-catenin. Afterward, β-catenin accumulates in the nucleus to activate members of the TCF and LEF families, which further modify target genes, subsequently promoting several processes including EMT [22]. The regulation of the close relation between β-catenin and EMT by hypotolide is verified in this study.

Hyptolide has been identified as the major active compound isolated from *Hyptis pectinata* (L.) Poit., and holds promise as a potential chemotherapeutic drug. However, the precise mechanism underlying the efficacy of hyptolide therapy remains unclear. This study aimed to elucidate the specific mechanisms underlying the therapeutic effects of hyptolide.

## Materials and methods

### Chemical

Hyptolide is a bioactive compound derived from the plant *Hyptis pectinata* collected from Ungaran, Central Java. The fresh leaves, stems, and twigs were extracted using the percolation method with ethanol as the solvent. The extract was concentrated using a rotary evaporator. Subsequently, distilled water was added to dissolve the chlorophyll, facilitating the separation of chlorophyll-free hyptolide. The concentrated solution was further processed using a rotary evaporator to produce a hyptolide precipitate, which was filtered through a Buchner funnel to yield hyptolide crystals. These crystals were purified via crystallization with ether until a high purity level was achieved using FTIR spectroscopy and HPLC assay. For experimental purposes, hyptolide was diluted in dimethyl sulfoxide (DMSO) to create stock solutions, which were further diluted to meet the required treatment concentrations before application.

### Cell line and culture condition

The cell line utilized in this study is the human ovarian cancer cell line IGROV1, cultured in Gibco Roswell Park Memorial Institute (RPMI) 1640 medium. The medium was supplemented with penicillin, streptomycin, and 10% fetal bovine serum. All cells were treated under controlled environmental conditions of 98% humidity, 5% CO_2_, and a temperature of 37 °C. Wild-type IGROV1 (IGROV1-WT) cells without cisplatin resistance were subjected to periodic cisplatin treatment, which resulted in the development of cisplatin resistance. IGROV1-CP1#1 and IGROV1-CP1#2 are IGROV1 cells resistant to 1 μM and 2 μM cisplatin, respectively.

### Cell proliferation assay

Cell proliferation was assessed using the CCK-8 assay. Hyptolide treatments were administered over varying periods at 37 °C in a 5% CO_2_ environment. The number of proliferating cells was determined by adding CCK-8 reagent (#3CK04-11, Dojindo Molecular Technologies, Inc.) according to the manufacturer's instructions. Absorbance was measured using an ELISA reader at a wavelength of 460 nm.

### Protein quantification and Western blotting assay

Cells subjected to various treatments were collected on ice using RIPA lysis buffer. The collected cell lysates were subjected to protein extraction by ultrasonic cell disruption. The extracted proteins were quantified using a Protein Assay Kit (#5,000,112; Bio-Rad). The proteins were denatured at 95 °C for 10 min. Protein separation was performed using 5–10% SDS/PAGE, and the proteins were transferred onto nitrocellulose membranes (#66,485, Pall). The membranes were blocked with 5% nonfat milk in Tris-buffered saline containing 0.1% Tween 20 (TBST) for 1 h at 25 °C, then incubated overnight at 4 °C with primary antibodies specific to the target proteins. After incubation, unbound antibodies were washed out of the membranes with TBST. The secondary antibody used were horseradish peroxidase-conjugated immunoglobins (Jackson ImmunoResearch Laboratories) and was incubated for 1 h. The immunocomplexes were visualized using chemiluminescence (ECL, PerkinElmer), and signal detection was performed using an Amersham Imager 600 system (GE Healthcare Life Sciences).

### Immunofluorescence staining

The cells were fixed and permeabilized in ice-cold PBS containing 4% paraformaldehyde and 0.5% Triton X-100, respectively. Then, cells were blocked using Invitrogen's CAS Blocking Histochemical Reagent for 1 h at room temperature, followed by overnight incubation with β-catenin primary antibodies (sc-7199, Santa Cruz). Cells were then incubated with Hoechst 33,342 dye for DNA binding and the corresponding Alexa Fluor 488-conjugated secondary antibody from Invitrogen at room temperature for 1 h. Fluorescence images were captured using an Olympus FLUOVIEW FV3000 confocal laser scanning microscope at excitation wavelengths of 405 nm and 488 nm. The fluorescence intensity and distribution were calculated using ImageJ software.

### Statistical analysis

Each analysis was performed with a minimum of three independent biological replicates, and the mean values were calculated along with the standard error of the mean (SEM). The collected data were statistically analyzed using either a t-test or a one-way analysis of variance conducted using SPSS Statistics 26. Significant differences are indicated by the following notations: *p < 0.05, **p < 0.01, and ***p < 0.001.

## Results

### Hyptolide treatment decreases ovarian cancer cell viability through the activation of ER stress pathways

**Fig. 1:**
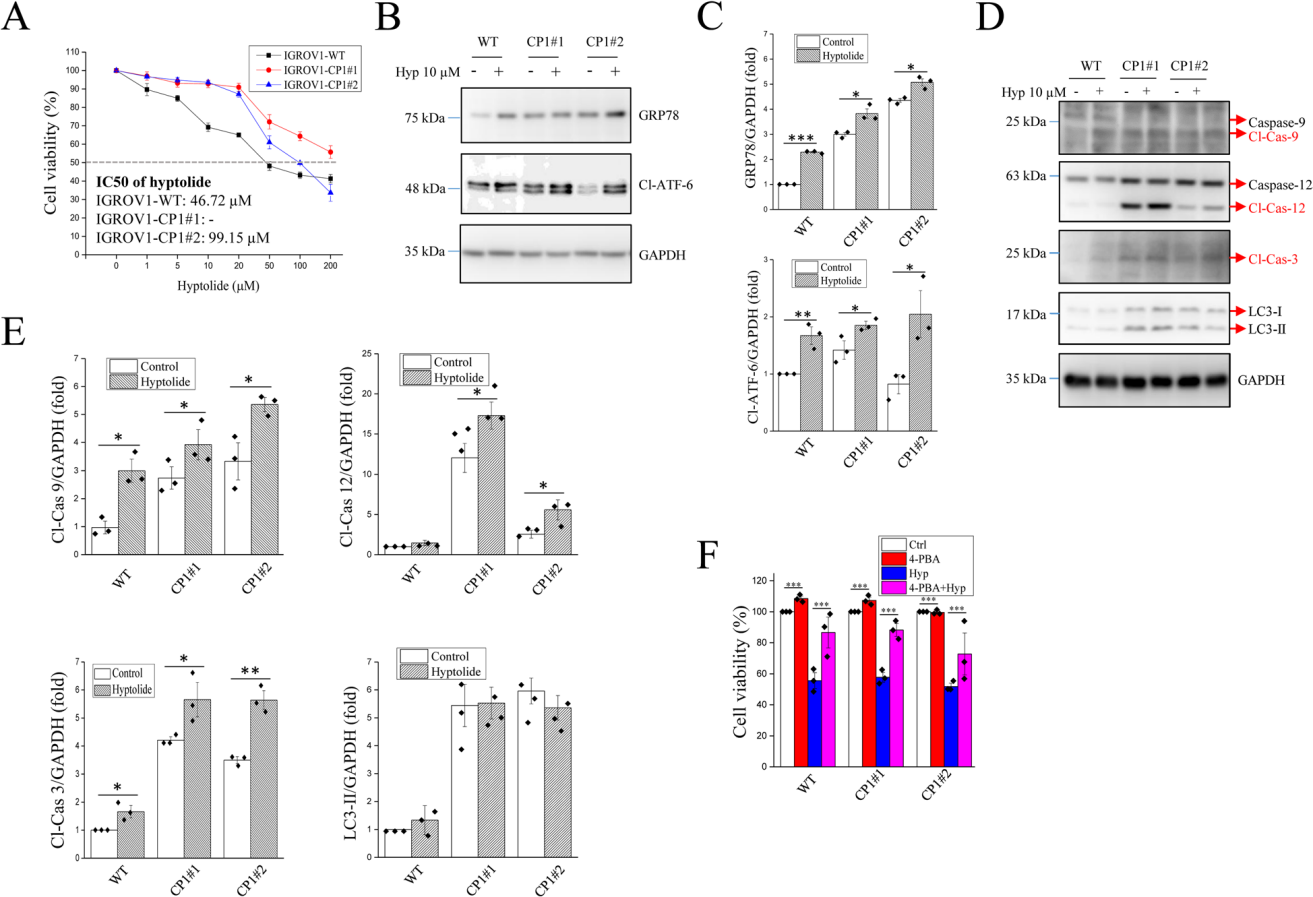
Hyptolide treatment decreases the viability of ovarian cancer cells. **A** Ovarian cancer cells (WT, CP1#1, and CP1#2) were treated with various concentrations of hyptolide for 48 h and then CCK8 reagent was added for 2 h. The cell viability was examined using ELISA Reader at a wavelength of 460 nm. **B**, **D** Representative blots and (**C**, **E**) quantification of GRP78, ATF-6, caspase-9, caspase-12, caspase-3, LC3 and GAPDH levels. **F** WT, CP1#1, and CP1#2 cells were treated with 10 μM hyptolide and 1 nM 4-PBA for 48 h. Cell viability was analyzed using a CCK8 assay. Analyses were conducted using three independent replicates with the bars representing SEM. Data were found to be significant at *p < 0.05, **p < 0.01 or ***p < 0.001

A cytotoxicity assay was performed to determine the response of ovarian cancer cells to hyptolide treatment. Wildtype (WT) and cisplatin-resistant (CP1#1 and CP1#2) ovarian cancer cells that had been incubated overnight were treated with hyptolide at various concentrations (0–200 µM) for 48 h. The results showed that WT cells were more sensitive to hyptolide, whereas CP1#1 and CP1#2 cells were more resistant to hyptolide treatment (Fig. 1A). Next, the increase in ER stress in cells after hyptolide treatment was compared with that in the non-treatment group (Fig. 1B). Overexpression of the upstream ER stress marker GRP78 and its activated downstream protein cleaved ATF-6 was observed following hyptolide treatment (Fig. 1C). In addition, cleavage and activation of ER stress-induced apoptosis-specific caspase-12, mitochondrial apoptosis-specific caspase-9 and the major effector caspase caspase-3 were higher in hyptolide-treated ovarian cancer cells. However, there was no difference in autophagic LC3 protein expression after hyptolide treatment (Fig. 1D, E). This indicates that hyptolide treatment may cause ER stress, resulting in mitochondrial dysfunction. However, hyptolide had no significant regulatory effect on the expression of the anti-apoptotic Bcl-2 and pro-apoptotic Bak proteins (Fig. S1). The ER stress inhibitor, 4-PBA, was used to verify the critical role of ER stress in hyptolide-induced cell death. The addition of 4-PBA inhibited hyptolide-induced GRP78 upregulation (Fig. S2) and reversed the cell death caused by hyptolide treatment in ovarian cancer cells (Fig. 1F). Therefore, ER stress may be the major cause of hyptolide-induced cell death.

### Hyptolide-induced GSK3β activation promotes cisplatin sensitivity in chemoresistant ovarian cancer cells

**Fig. 2:**
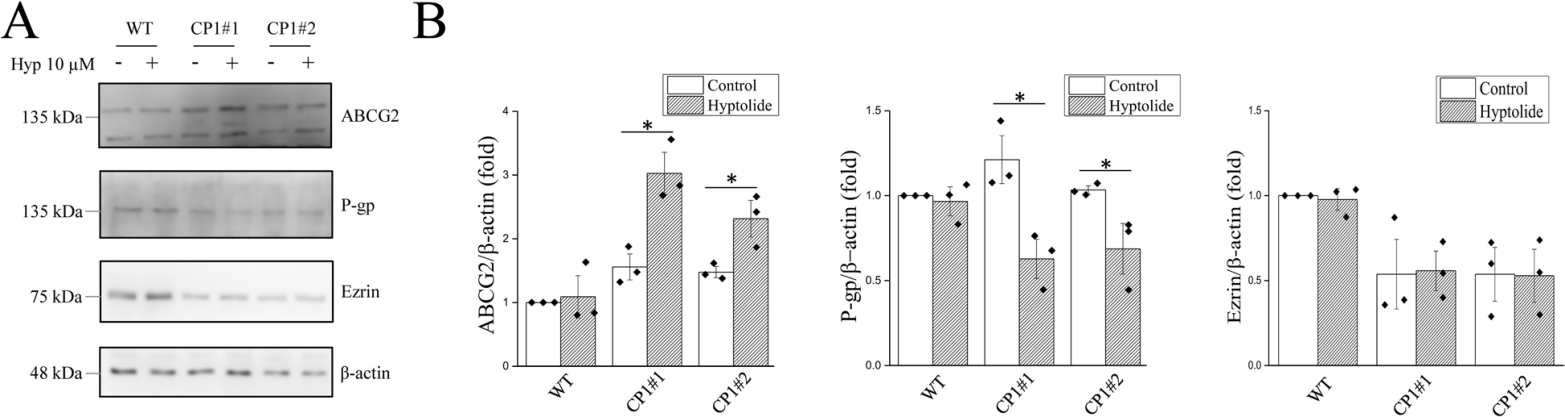
Hyptolide regulates transporters for cisplatin transportation. Cisplatin is taken up and excreted by cells using ABCG2 and P-gp transporters, respectively. WT, CP1#1, and CP1#2 cells were treated with 10 μM hyptolide for 48 h. **A** Representative blots and **B** quantification of ABCG2, P-gp, Ezrin, and β-actin levels. Analyses were conducted using three independent replicates with the bars representing SEM. Data were found to be significant at *p < 0.05

The cisplatin-resistant ovarian cancer cells (CP1#1 and CP1#2) were more resistant to hyptolide treatment (Fig. 1A). ABCG is a drug influx transporter that increases the number of compounds that can enter the cells. P-gp is a drug efflux transporter that promotes the release of drug compounds from cells. Ezrin is a member of the ezrin–radixin–moesin protein family, which contributes to chemotherapy resistance. Increased ABCG2 expression but decreased P-gp expression was observed in CP1#1 and CP1#2 cells, but not in WT cells after hyptolide treatment (Fig. 2). However, hyptolide treatment did not affect the ezrin levels (Fig. 2). The above results suggest that hypotolide increases the reversion of cisplatin-chemoresistant cells to cisplatin-chemosensitive cells.

**Fig. 3:**
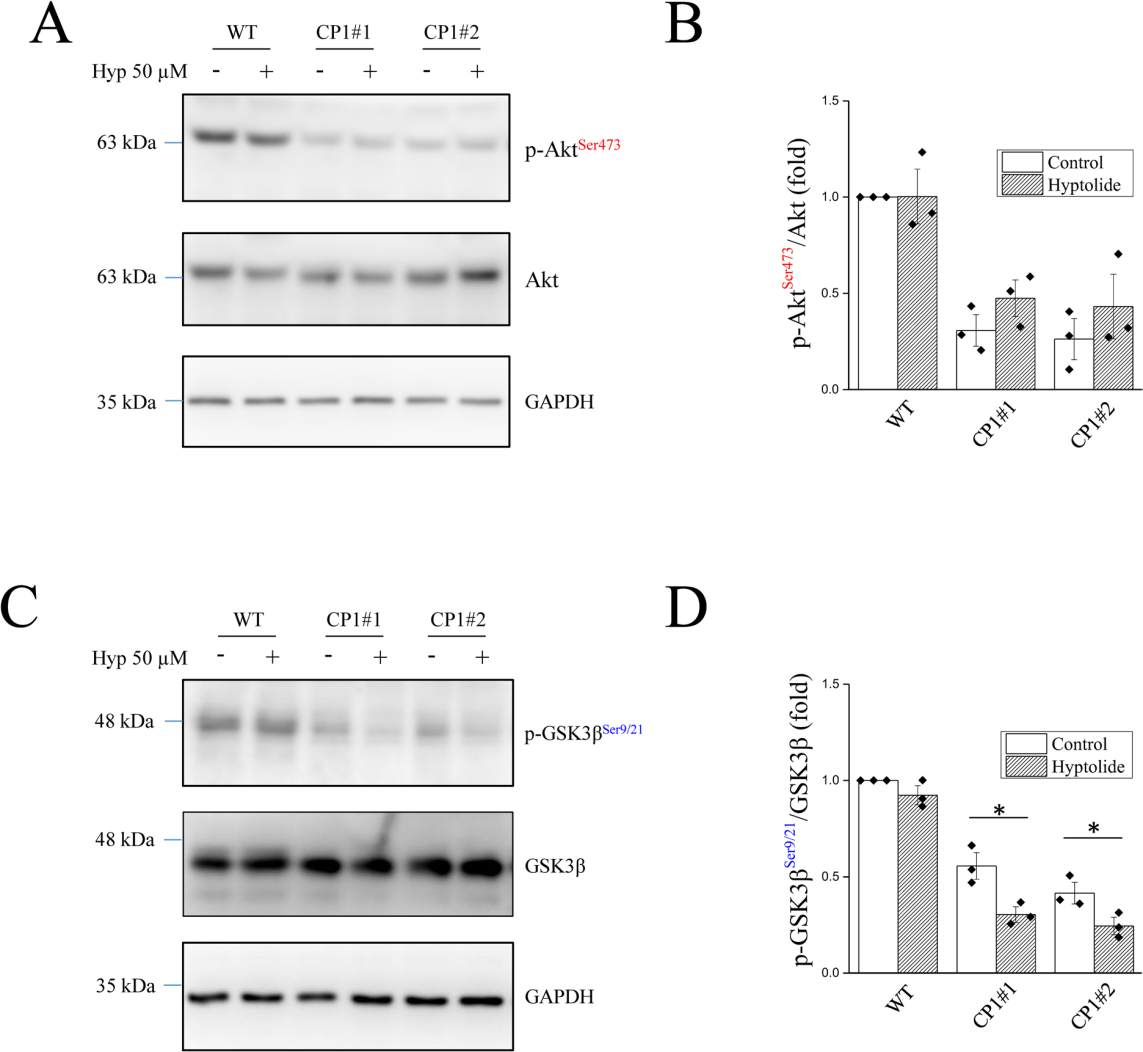
Hyptolide treatment activates GSK3β but not Akt. WT, CP1#1, and CP1#2 cells were treated with 50 μM hyptolide for 30 min. **A**, **C** Representative blots and (**B**, **D**) quantification of phosphorylated Akt (p-Akt^Ser473^), Akt, phosphorylated GSK3β (p-GSK3β^Ser9/21^), GSK3β, and GAPDH. Analyses were conducted using three independent replicates with the bars representing SEM. Data were found to be significant at *p < 0.05

The PI3K/Akt/GSK-3β pathway is a significant cause of chemoresistance in cancer therapy. The phosphorylation of Akt at Ser467 activates Akt in the PI3K/Akt pathway. Hyptolide treatment did not affect Akt activation in WT or cisplatin-resistant cells (CP1#1 and CP1#2) (Fig. 3A, B). On the other hand, hyptolide treatment decreased GSK3β phosphorylation at Ser9/21 (Fig. 3C, D). This indicates an hyptolide-induced increase in GSK3β activity. Interestingly, this phenomenon was observed in CP1#1 and CP1#2 cells, but not in WT cells.

### Hyptolide promotes mesenchymal-to-epithelial transition (MET) progression by inhibiting β-catenin nuclear translocation

**Fig. 4:**
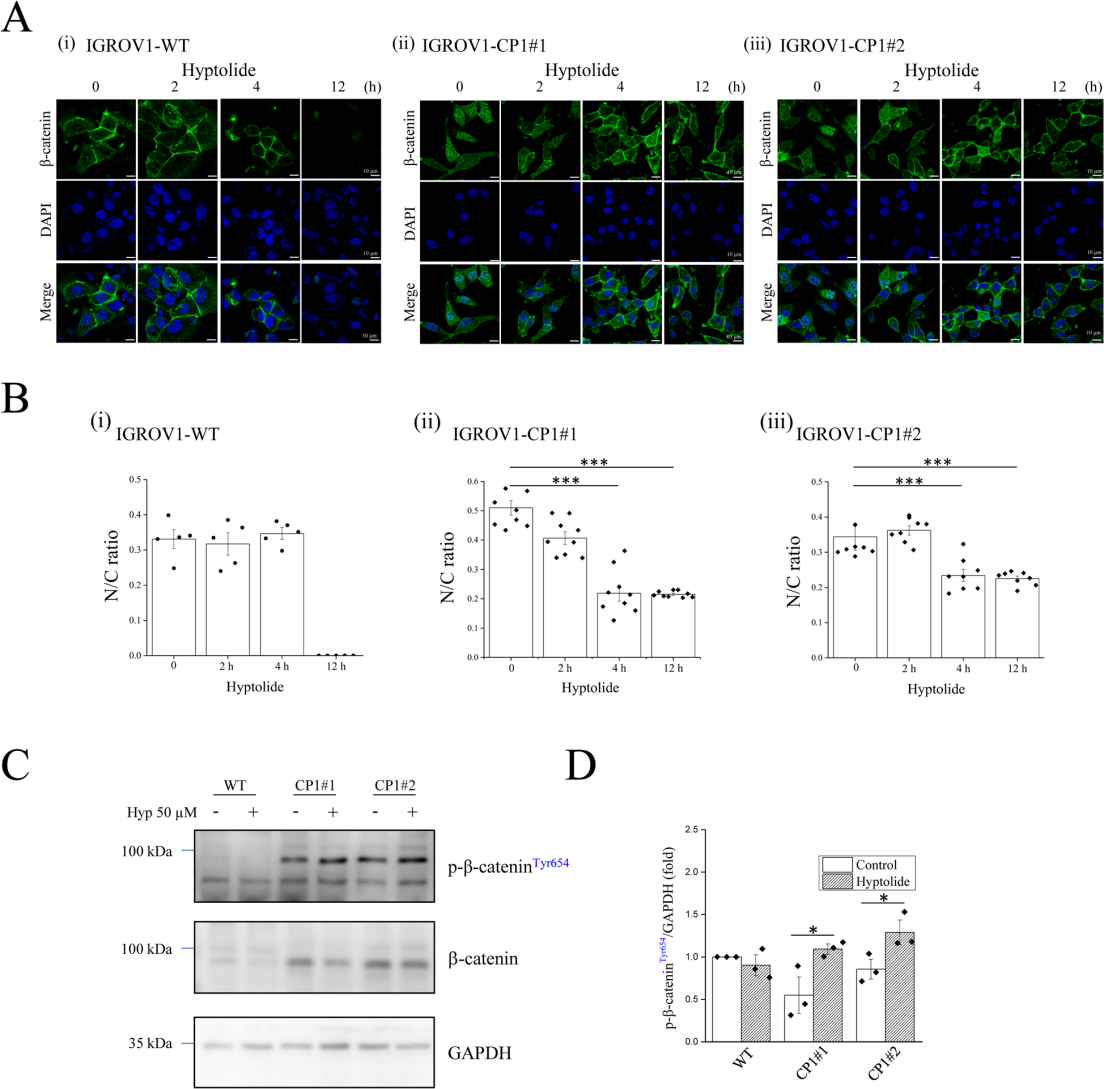
Hyptolide inhibits nuclear translocation of β-catenin in cisplatin-resistant ovarian cancer cells. WT, CP1#1, and CP1#2 cells were treated with 10 μM hyptolide for 0, 2, 4, and 12 h. **A** Immunofluorescence staining of β-catenin (green) and DAPI nuclear stain (blue) was performed and fluorescence images were captured using confocal microscopy (FV3000, OLYMPUS). **B** Quantification of the nuclear and cytoplasmic fluorescence intensities of β-catenin. The nuclear–cytoplasmic ratio (N/C ratio) of β-catenin was quantified and calculated using ImageJ software. (**C**) Representative blots and (**D**) quantification of phosphorylated β-catenin (p-β-catenin^Tyr654^), β-catenin, and GAPDH. Analyses were conducted using three independent replicates with the bars representing SEM. Data were found to be significant at *p < 0.05, ***p < 0.001

β-catenin is located downstream of the PI3K pathway and plays an important role in chemoresistance in cancer cells. To determine the effect of hyptolide on β-catenin expression, we found that cisplatin-resistant CP1#1 and CP1#2 cells had higher β-catenin expression levels than WT cells (Fig. 4C). Immunofluorescence staining showed that β-catenin in WT cells was mainly distributed near the cell membrane. However, β-catenin in cisplatin-resistant CP1#1 and CP1#2 cells was mainly distributed near the nucleus and in the cytoplasm (Fig. 4A). Hyptolide treatment inhibited the nuclear translocation of β-catenin in CP1#1 and CP1#2 cells and redistributed it to the cell membrane. However, hyptolide did not affect the distribution of β-catenin in WT cells, and prolonged hyptolide treatment (12 h) decreased β-catenin expression (Fig. 4A, B). Phosphorylation at Tyr654 inactivates β-catenin. The phosphorylation of β-catenin Tyr654 in WT cells was higher than that in CP1#1 and CP1#2 cells under resting conditions (Fig. 4C, D). In cisplatin-resistant cells, hyptolide increased the phosphorylation of β-catenin at Tyr654, which decreased β-catenin activation (Fig. 4C, D). However, the inhibitory effect of hyptolide on β-catenin activity was not observed in WT cells.

**Fig. 5:**
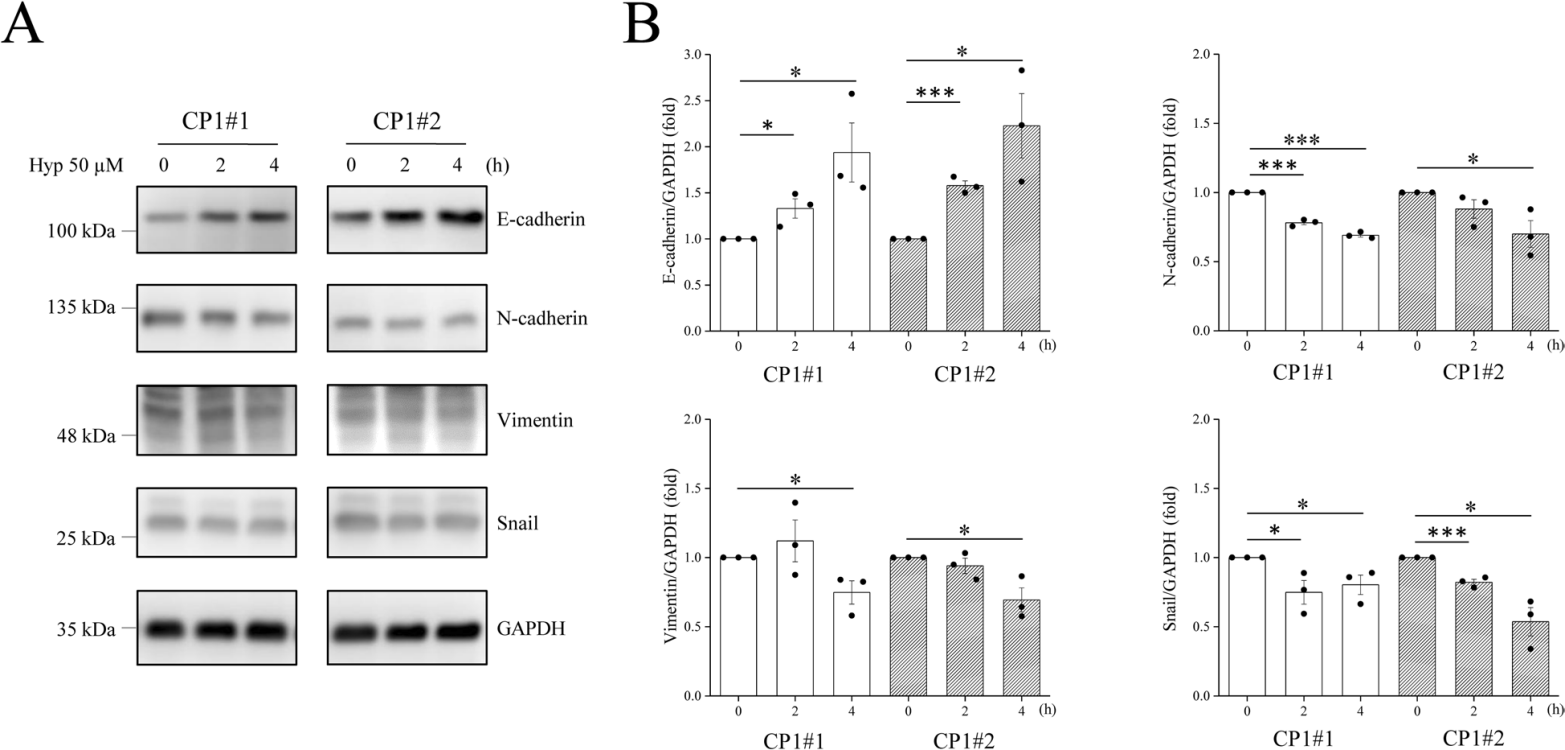
Hyptolide promotes mesenchymal-to-epithelial transition in cisplatin-resistant cells. CP1#1 and CP1#2 cells treated with 50 μM hyptolide for 0, 2 and 4 h. **A** Representative blots and (**B**) quantification of E-cadherin, N-cadherin, vimentin, Snail and GAPDH levels. Analyses were conducted using three independent replicates with the bars representing SEM. Data were found to be significant at *p < 0.05 or ***p < 0.001

It has been previously reported that β-catenin activation induces the progression of EMT, a process opposite to MET, that permits a decrease in intercellular adhesion and enhances cell migration. MET plays an important regulatory role in reversing chemoresistance in cancer cells. Increasing the expression of cell adhesion protein E-cadherin and inhibiting the nuclear entry of β-catenin can be regarded characteristic of MET. Hyptolide treatment increased the expression of the epithelial marker E-cadherin and downregulated the expression of the mesenchymal markers N-cadherin and vimentin, as well as the EMT transcription factor Snail in cisplatin-resistant CP1#1 and CP1#2 cells over time (Fig. 5A, B). On the other hand, LiCl, an inhibitor of GSK3β, effectively inhibited the increase in E-cadherin expression induced by hyptolide (Fig. S3).

### Combination treatment with hyptolide and cisplatin decreases cisplatin-resistant ovarian cancer cell viability

**Fig. 6:**
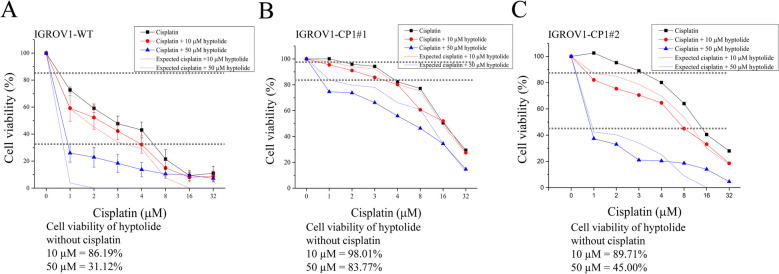
Synergistic effect of combination therapy with hyptolide and cisplatin in cisplatin-resistant ovarian cancer cells. **A** WT, (**B**) CP1#1, and (**C**) CP1#2 cells were treated with hyptolide (10 µM and 50 µM) and different concentrations of cisplatin for 48 h. Cell viability was analyzed using CCK8 assay. Analyses were conducted using three independent replicates

Our study was based on the hypothesis that combination therapy with hyptolide and cisplatin could reverse chemoresistance in cisplatin-resistant cells. The expected cell survival rate was the sum of the cell death rates caused by cisplatin and hyptolides. If the cell death rate caused by the combination therapy of cisplatin and hyptolide was greater than the expected cell survival rate, we considered this to be an additive cytotoxic effect. Based on the results of cytotoxic cell analysis, combination therapy with hyptolide and cisplatin did not increase the mortality of WT cells (Fig. 6A). However, in cisplatin-resistant CP1#1 and CP1#2 cells, the actual survival rates after cisplatin and hyptolide combination therapy were lower than expected (Fig. 6B, C). This indicated that combination therapy with hyptolide and cisplatin can increase the effectiveness of cisplatin therapy in cisplatin-resistant cells.

## Discussion

Hyptolide has been studied in several chemoresistant cancer cell lines [12]. Our results confirmed that hyptolide suppressed the growth of wild-type and chemoresistant ovarian cancer cell lines. However, effective results in cisplatin-resistant cells requires the use of relatively high hyptolide concentrations. Therefore, we attempted to use a combination therapy of hyptolides and cisplatin.

A Fourier transform infrared (FTIR) graph was used to identify hyptolide and its functional groups (Fig. S4A). The FTIR image of hyptolide shows the highest absorption peak at a wavelength of 1740 cm^−1^ which corresponds to a C = O lactone or ester stretching absorption [14]. The HPLC assay shows a single sharp peak at a retention time of approximately 2.27 min, indicating that the hyptolide used has high purity (Fig. S4B). In previous studies, both natural and synthetic lactones were shown to possess antitumor activity [23]. For example, α,β unsaturated δ- lactone suppresses cancer cell growth [13, 24] through induction of ER stress [25]. Various lactone compounds trigger apoptosis mediated by ER stress [26, 27]. In this study, we found an increase in GRP78 and ATF6 ER stress markers in both wild-type and chemoresistant ovarian cancer cell lines (Fig. 1B). ER stress often leads to mitochondrial dysfunction. Excessive or long-term ER stress may induce apoptosis [28]. Indeed, hyptolide activated the apoptosis-related proteins caspase-9, caspase-12 and caspase-3 (Fig. 1D). This indicates that ER stress and mitochondrial dysfunction are involved in hyptolide-induced apoptosis. The ER stress inhibitor 4-PBA reduced the cytotoxic effects of hyptolide (Fig. 1F). Additionally, hyptolide treatment increased ABCG2 expression and reduced P-gp expression in cisplatin-resistant CP1#1 and CP1#2 cells, which is a possible therapeutic mechanism for overcoming drug-resistant cancer cells (Fig. 2).

As there is no clear cytotoxic mechanism for hyptolide, we focused on the lactone group in hyptolide, which triggers the PI3K/Akt pathway [29]. Thus, we examined the PI3K pathway proteins including Akt, GSK3β, and β-catenin. Our results showed no difference in Akt activity after hyptolide treatment (Fig. 3A, B). However, increased GSK3β activation was observed in chemoresistant ovarian cancer cells with hyptolide treatment (Fig. 3C, D).

Nuclear translocation of β-catenin plays a key role in EMT. β-catenin is a transcription factor, which has been shown to activate LAF-1 transcription and regulates genes related to EMT [30]. In chemoresistant ovarian cancer, it is known that β-catenin is present in the nucleus [31]. β-catenin was mainly distributed in the cell membrane of wild-type cells, whereas it was mainly present in the cytoplasm and nucleus of cisplatin-resistant CP1#1 and CP1#2 cells (Fig. 4). This difference in cellular location supports the possibility that β-catenin may be involved in cisplatin resistance and EMT progression of ovarian cancer cells. The translocation of β-catenin from the nucleus to the cell membrane and cytoplasm with hyptolide treatment in cisplatin-resistant CP1#1 and CP1#2 cells can convert cisplatin chemoresistance to cisplatin chemosensitivity during MET (Fig. 4). Restoration of E-cadherin expression can reduce metastasis and resistance to chemotherapeutic drugs by promoting MET progression [32]. Our experimental data support the idea that hyptolide increases the expression of E-cadherin but decreases the expression of N-cadherin, vimentin and Snail in cisplatin-resistant CP1#1 and CP1#2 cells (Fig. 5). Additionally, previous studies have reported that GSK3β inhibits EMT to reduce cancer cell metastasis and progression [33] that related to our study.

## Conclusion

**Fig. 7:**
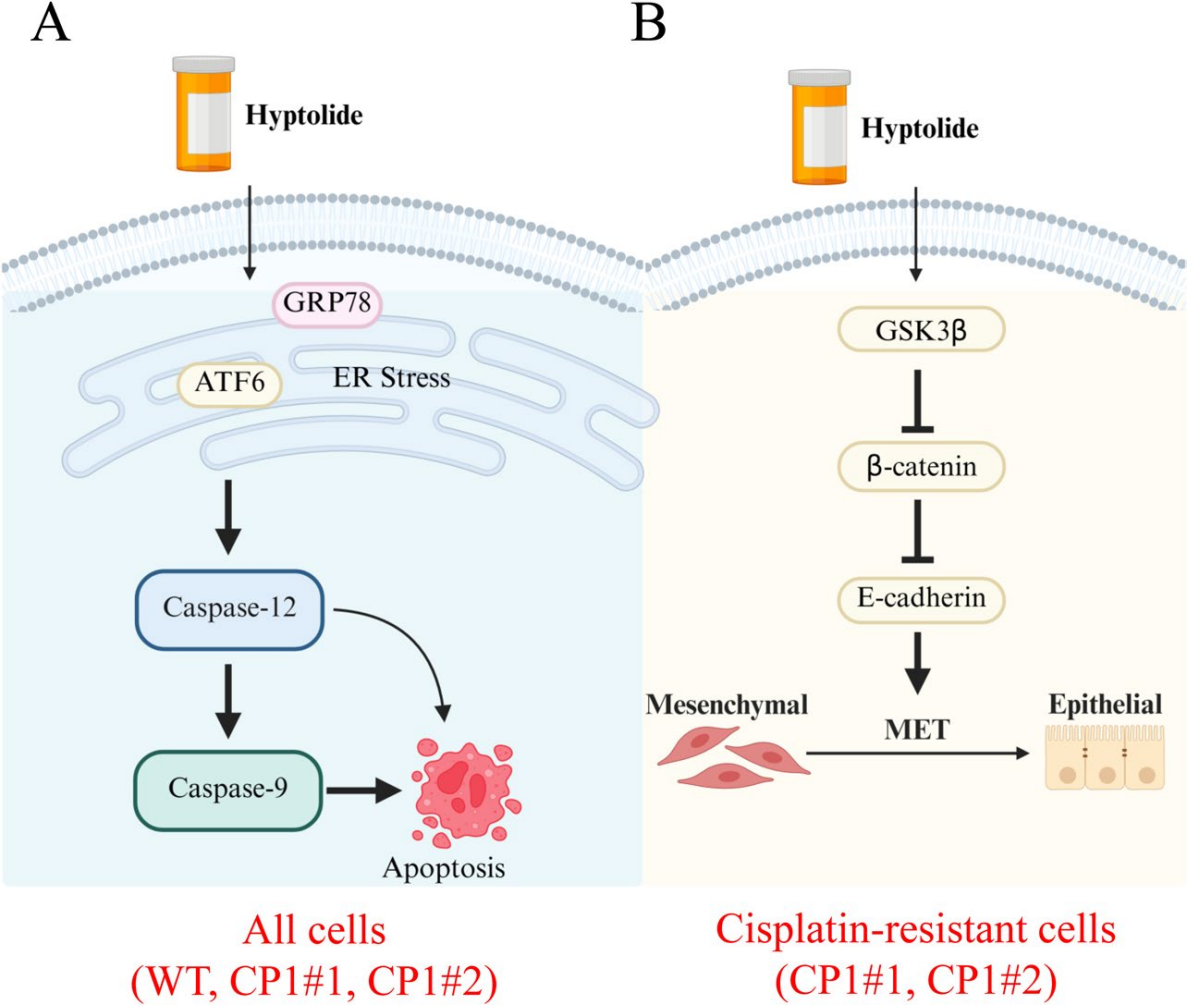
Schematic of the molecular mechanism of hyptolide acting on ovarian cancer cells. **A** Hyptolide induces cell apoptosis in WT and cisplatin-resistant ovarian cancer cells through induction of ER stress. **B** Hyptolide inhibits the activity of β-catenin and increases the expression of E-cadherin by activating GSK3β in cisplatin-resistant ovarian cancer cells

In summary, hyptolide induces apoptosis in ovarian cancer cells by inducing ER stress. In particular, hyptolide inhibits the activity of β-catenin and increases the expression of E-cadherin by activating GSK3β in cisplatin-resistant ovarian cancer cells. Inhibiting the progression of EMT not only inhibits cancer cell metastasis, but also increases the sensitivity of chemoresistant cancer cells to chemotherapy (Fig. 7). Therefore, hyptolide has the potential to be used in the treatment of cancer.

## Supplementary Information

Supplementary Material 1. Fig. S1 Bcl-2 family proteins are not involved in hyptolide treatment. Ovarian cancer cells (WT, CP1#1, and CP1#2) were treated with 10 μM hyptolide for 24 h. (A) Representative blots and (B) quantification of Bcl-2, Bak, and GAPDH. Analyses were conducted from three independent replicates with the bars representing SEM.

Supplementary Material 2. Fig. S2 4-PBA inhibits hyptolide-induced ER stress activation. Ovarian cancer cells (WT, CP1#1, and CP1#2) were treated with 10 μM hyptolide and 1 nM 4-PBA for 24 h. Representative blots of GRP78 and GAPDH from three independent replicates.

Supplementary Material 3. Fig. S3 GSK3β inhibitor, LiCl, reduces the hyptolide-induced increase in E-cadherin levels. Ovarian cancer cells (WT, CP1#1, and CP1#2) were treated with 50 μM hyptolide and 10 μM LiCl for 30 min. (A) Representative blots and (B) quantification of E-cadherin and GAPDH. Analyses were conducted using three independent replicates with the bars representing SEM. Data were found to be significant at *p<0.05 or **p<0.01.

Supplementary Material 4. Fig. S4 Purity analysis of hyptolide using FTIR and HPLC. (A) Graphic profile of crystal hyptolide analyzed using FTIR spectrometer. (B) The chromatogram determined by HPLC showed a single sharp peak at a retention time of approximately 2.27 minutes.

## Data Availability

No datasets were generated or analysed during the current study.

## Abbreviations

ER: Endoplasmic reticulum
WT: Wild-type
GRP78: Glucose-regulated protein 78
ATF-6: Activating transcription factor 6
LC3: Microtubule-associated protein 1 light chain 3
ABCG2: ATP-binding cassette subfamily G member 2
P-gp: P-glycoprotein
PI3K: Phosphatidylinositol 3-kinase
GSK3β: Glycogen synthase kinase 3 beta
MET: Mesenchymal-epithelial transition
EMT: Epithelial-mesenchymal transition
